# Radiofrequency ablation for treatment of locally recurrent thyroid cancer presenting as a metastatic lymph node with dense macrocalcification: A case report and literature review

**DOI:** 10.1097/MD.0000000000010003

**Published:** 2018-03-02

**Authors:** Roh-Eul Yoo, Ji-hoon Kim, Jin Chul Paeng, Young Joo Park

**Affiliations:** a Department of Radiology, Seoul National University College of Medicine, Seoul, Korea; b Department of Nuclear Medicine; c Department of Internal Medicine, Seoul National University Hospital, Seoul, Korea.

**Keywords:** dense macrocalcification, lymph node, papillary thyroid carcinoma, radioactive iodine, radiofrequency ablation

## Abstract

**Rationale:**

Long-term recurrence rate of differentiated thyroid carcinoma has been reported to be as high as 30%. Repeat surgery may be challenging due to normal tissue plane distortion secondary to postoperative fibrosis, especially for small-sized recurrences. Recently, radiofrequency ablation (RFA) has been suggested to be a safe and effective alternative for high-risk patients or those who refuse surgery. Nonetheless, the efficacy of RFA remains questionable for densely calcified lymph nodes, which would have an increased likelihood of leaving residues after RFA.

**Patient concerns:**

We present a case of a successful combined treatment of a metastatic lymph node with dense macrocalcification with the use of a single RFA session and radioactive iodine (RAI) ablation in a patient with a previous history of total thyroidectomy and neck node dissection for papillary thyroid carcinoma.

**Diagnoses:**

A 71-year-old man with papillary thyroid carcinoma underwent total thyroidectomy and neck node dissection followed by RAI ablation. The stimulated serum thyroglobulin level was 4.74 ng/mL at the time of RAI ablation, and the follow-up ultrasonography 3 months later revealed a 15-mm lymph node with dense macrocalcification at the right cervical level III.

**Interventions:**

After confirming metastasis on cytology, the lesion was treated with ultrasound-guided RFA.

**Outcomes:**

The single RFA session combined with RAI ablation led to biochemical remission at 5 months after RFA, and complete resolution of structural recurrence including macrocalcification was observed 7 months after the second RAI (1 year after RFA). The patient remained free of recurrence at the 5-year follow-up.

**Lessons:**

RFA may offer a safe and effective alternative to ‘berry picking’ surgery in cases of surgical ineligibility or patient refusal of surgery even when the target lesions contain dense macrocalcification.

## 1 Introduction

Despite the low mortality rate of approximately 0.5 deaths per 100,000 people, the long-term recurrence rate of differentiated thyroid carcinoma has been reported to be as high as 30%.^[1–3]^ Although repeat surgery has been regarded as the treatment of choice to improve long-term survival in the majority of recurring tumors, normal tissue plane distortion secondary to postoperative fibrosis can pose a challenge to surgeons and predispose patients to debilitating conditions and postoperative complications, such as hoarseness and hypocalcemia, thus resulting in an unfavorable benefit-to-risk profile, especially for small-sized recurrences.^[1,4,5]^

Therefore, the American Thyroid Association guidelines suggest that active surveillance be employed for small lymph nodes.^[6]^ However, in routine clinical practice, it is not always easy for patients with detectable serum thyroglobulin at follow-up to make the decision to observe the lesions on a regular basis once metastasis is confirmed by fine-needle aspiration (FNA) or core needle biopsy.

Recently, percutaneous ethanol injection therapy (PEIT) and thermal ablation such as radiofrequency ablation (RFA) have been suggested to be safe and effective alternatives for high-risk patients or those who refuse surgery.^[7–17]^ Nonetheless, metastatic lymph nodes in thyroid cancers are known to accompany calcification more frequently than those in other cancers, and the efficacy of RFA remains questionable for densely calcified lymph nodes which would have an increased likelihood of leaving residues after RFA.

Herein, we present a case of a successful combined treatment of a metastatic lymph node with dense macrocalcification with the use of a single RFA session and radioactive iodine (RAI) ablation in a patient with a previous history of total thyroidectomy and neck node dissection for papillary thyroid carcinoma.

## 2 Case report

This study was approved by the institutional review board of our hospital, and the requirement for informed consent was waived due to its retrospective nature.

A 71-year-old man was diagnosed with the follicular variant papillary thyroid carcinoma in the right thyroid gland after total thyroidectomy with a bilateral central lymph node and modified radical neck dissection. The tumor was pathologically staged as pT3N1b according to the American Joint Committee on Cancer (AJCC, 7th edition) TNM staging system due to extrathyroid extension and lymph node metastases at the right cervical level VI, right level II, right level IV, and left level II, according to the level system defined by the American Head and Neck Society.

**Figure 1:**
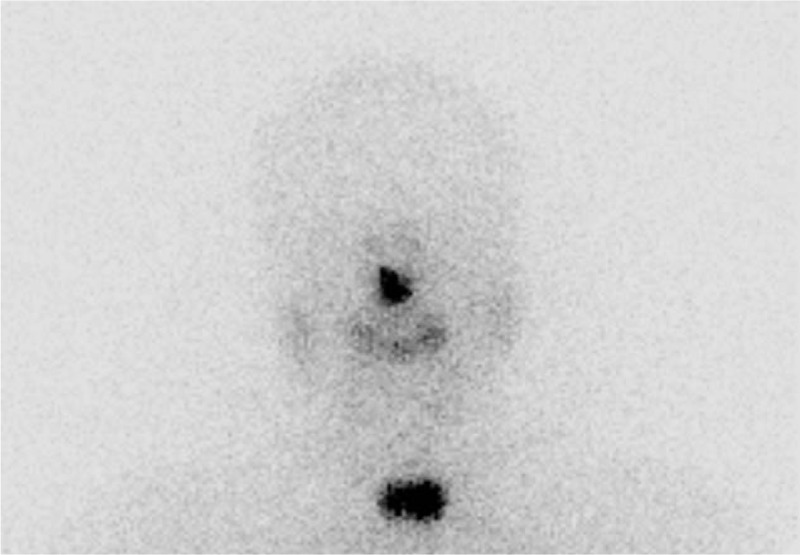
Whole body scan after rhTSH-stimulated RAI. Remnant uptake was observed only at the thyroid bed.

Three months after the surgery, the patient received remnant ablation therapy using I-131 30 mCi with recombinant human thyrotropin (rhTSH). On the whole body scan after rhTSH-stimulated RAI, remnant uptake was observed at the thyroid bed (Fig. 1), and the rhTSH-stimulated serum thyroglobulin level was 4.74 ng/mL.

**Figure 2:**
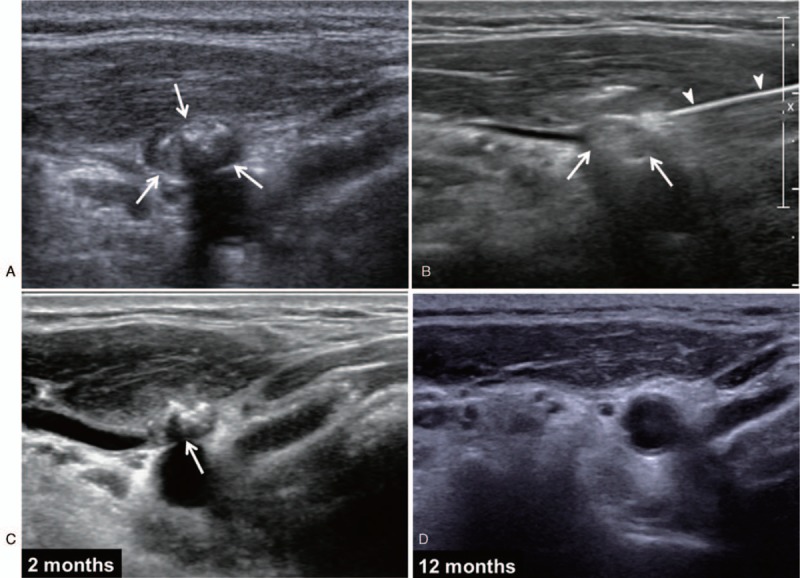
Axial US image (A) shows an approximately 15 mm ovoid lymph node with dense macrocalcification at the right level III (arrows) noted on follow-up US examination performed 6 months after the surgery. (B) After local anesthesia, a radiofrequency electrode (arrows) was inserted into the lymph node (arrowheads). (C) The 2-month follow-up US revealed a volume reduction of 43% (arrow). (D) A complete resolution was achieved at the 12-month US follow-up.

**Figure 3:**
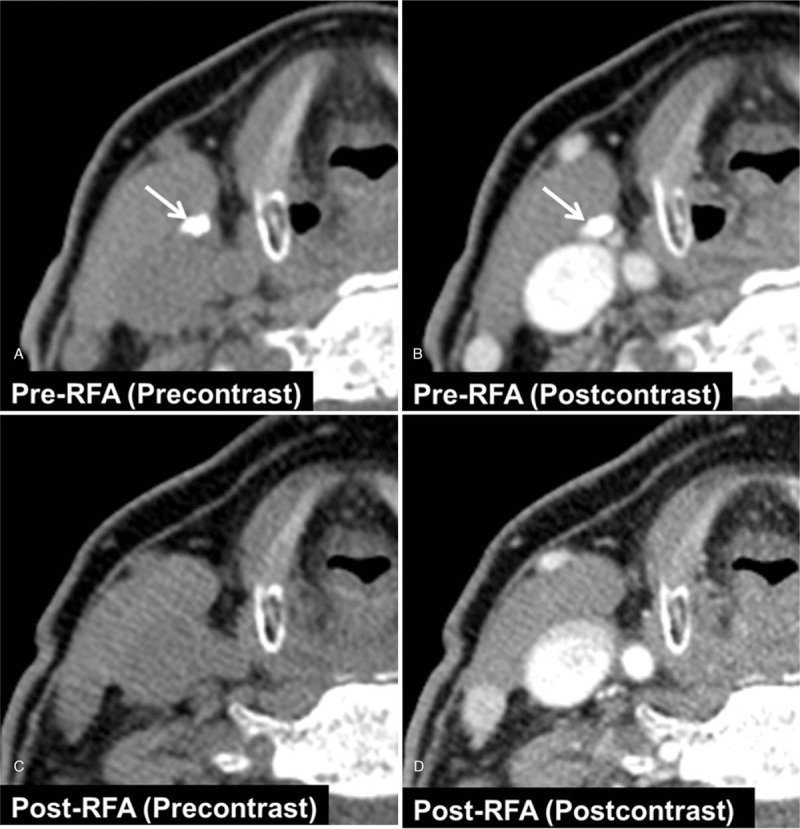
Pre- and post-RFA thyroid CT. (A, B) Pre-RFA CT shows a suspicious lymph node with dense macrocalcification at the right level III (arrow). (C, D) Follow-up thyroid CT performed 3 years after RFA shows no demonstrable residual lesion.

Follow-up ultrasonography (US) examination performed 6 months after the surgery revealed an ovoid lymph node at the right level III. The maximum short and long diameters were measured as 5 mm and 8 mm, respectively, on the transverse plane, and a maximum long diameter was 15 mm on the longitudinal plane. The lymph node showed an echogenicity higher than that of the strap muscles and contained dense macrocalcification in its central portion (Fig. 2A, arrows), which was also well depicted on thyroid CT (Fig. 3 A and 3B, arrow). Metastatic papillary carcinoma was confirmed on US-guided FNA.

After careful discussion with a team consisting of a surgeon, an endocrinologist, and a radiologist, the patient chose to undergo RFA rather than reoperation or active surveillance. A radiologist (J.H.K., 8 years of experience performing thyroid US imaging) performed RFA for the calcified metastatic lymph node, using a real-time US system (IU22; Philips Healthcare, Andover, Mass) equipped with a 12-MHz linear transducer and a RF generator (Radionics Cool-tip; Integra, Burlington, Mass). After local anesthesia with 2% lidocaine, a 7-cm-long 18-gauge internally cooled electrode with a 0.5-cm active tip (Well-point Radiofrequency Electrode; Taewoong Medical, Goyang, Korea) (Fig. 2B, arrowheads) was inserted into the lymph node (Fig. 2B, arrows). At first, ablation was performed in a unit-by-unit manner mainly at the periphery of the central macrocalcification using 30–40 W of power. Subsequently, attempts were made to advance the needle tip into the central portion with dense macrocalcification and further ablation was performed in a unit-by-unit manner. Real-time monitoring was performed with US imaging to ascertain correct needle positioning throughout the procedure. The total ablation time and needling time were 8 minutes and 45 minutes, respectively. No acute complications were noted after the procedure.

The lymph node showed a volume reduction of 43% at the 2-month follow-up US (Fig. 2C, arrow) and 68% at the 6-month follow-up. A complete resolution of structural recurrence including macrocalcification was achieved at the 12-month US follow-up (Fig. 2D). On the whole body scan after the second RAI ablation which was performed 5 months after the RFA, no remnant or abnormal uptake was observed, and the rhTSH-stimulated serum thyroglobulin level was 0.15 ng/mL. The rhTSH-stimulated serum thyroglobulin level was below 0.1 ng/mL even at the 17-month follow-up. Thyroid CT performed 3 years after RFA revealed no demonstrable residual lesion (Fig. 3 C and 3D). The patient remained recurrence-free at the 5-year follow-up.

## 3 Discussion

In the presented case, a single RFA session (performed in place of reoperation) led to the complete resolution of a structural recurrence manifesting as a lymph node with dense macrocalcification. The biochemical remission was also achieved after RFA and combined RAI ablation.

RFA has gained widespread use as an alternative to surgery for solid malignancies including the liver, kidneys, and lungs.^[18–20]^ During the procedure, a RF generator is used to produce voltage between an active electrode (applicator) and a reference electrode (grounding pad). The voltage is used to establish an oscillating electric field, which in turn induces frictional heating by causing electrons to collide with adjacent molecules closest to the applicator.^[21]^ Tissue heating to temperatures greater than 60°C causes immediate cell death.^[22]^

Over the past few years, there has been growing interest in the safety and efficacy of RFA for treating recurrent thyroid cancers.^[7–10,12,14,15,17]^ Baek et al. ^[7]^ have reported a significant volume reduction of approximately 90% after RFA in 12 local recurrences at either the operative bed or lateral neck nodes. Dysphonia occurred in one patient immediately after RFA. In another study that explored the role of RFA in patients with inoperable symptomatic recurrent thyroid cancers,^[15]^ a mean volume reduction of 50.9% was observed in 13 of 15 cases with resultant symptom relief in seven patients. No major complications were reported, with the exception of one case of skin burn. Lim et al.^[12]^ investigated the efficacy and safety of RFA in a larger number of cases and demonstrated that RFA resulted in a volume reduction of 95% in 61 treated cases, with complete resolution in 82% (50 of 61). Three patients complained of a transient voice change. With regard to the long-term efficacy of RFA, Monchik et al.^[14]^ have shown that 14 of 16 patients who were treated with RFA for either the operative bed or lateral neck recurrence remained disease-free at a mean follow-up of 40.7 months. One case of minor skin burn and one case of permanent vocal cord paralysis occurred as complications. More recently, Kim et al.^[9]^ have compared the safety and efficacy of RFA for localized small recurrent thyroid cancers less than 2 cm with those of repeat surgery. In this study, the 1- and 3-year recurrence-free survival rates and post-treatment hoarseness rates were comparable between the two groups, whereas post-treatment hypocalcemia occurred exclusively in the reoperation group.

The present case demonstrated the long-term efficacy of RFA for treatment of a small metastatic lymph node with dense central macrocalcification. In general, dense macrocalcification may be a major obstacle to various thyroid interventions including FNA and core needle biopsy given the technical difficulty in needling.^[23]^ Indeed, severe calcification was the cause of RFA treatment failure in one case in a previous study.^[15]^ In the present case, we not only performed ablation mainly at the periphery of the central macrocalcification but also attempted to penetrate the needle into the central portion with dense macrocalcification for further ablation. As a result, negative conversion of the stimulated serum thyroglobulin level as well as complete resolution on US imaging were achieved at follow-up. Biochemical remission may be attributed to the combined effect of RAI ablation and RFA. On the other hand, complete resolution of structural recurrence including macrocalcification is likely to be mainly due to RFA, given that no abnormal uptake was observed at the metastatic lymph node on the whole body scan after the first RAI treatment.

As compared with RFA, PEIT has the benefits of reduced cost, pain, and risk of developing nerve injury.^[9,14]^ However, results of the previous study comparing the efficacy of the two treatment modalities for hepatocellular carcinoma have suggested that RFA may be more effective in reducing the volume and thus require fewer treatment sessions for therapeutic success (volume reduction >50%) because it can produce a larger zone of tumor destruction than PEIT.^[14,24]^ Local tumor recurrence rates of 0–25% and 3.2–33% have been reported after RFA and ethanol ablation of recurrent thyroid cancers, respectively.^[16]^

Given its high efficacy and safety, RFA is viewed as an attractive alternative treatment option to ‘berry picking’ surgery. However, RFA is inherently limited by its ability to eradicate occult recurrent tumors that are invisible on US and those located in deep areas that are inaccessible to US. Moreover, large or numerous tumors may necessitate multiple RFA sessions. Hence, special care needs to be taken in deciding to perform RFA instead of repeat surgery for local control of recurrent tumors.

## 4 Conclusion

We have presented a case of recurrent papillary thyroid carcinoma manifesting as a metastatic lymph node with dense macrocalcification in which a single RFA session combined with RAI ablation led to complete resolution (in terms of both imaging and biochemical findings) and no recurrence at the 5-year follow-up. The present case demonstrated that RFA may offer a safe and effective alternative to ‘berry picking’ surgery in cases of surgical ineligibility or patient refusal of surgery even when the target lesions contain dense macrocalcification, provided that it is performed by an experienced radiologist.
